# Epigenetic modifications in early stage lung cancer: pathogenesis, biomarkers, and early diagnosis

**DOI:** 10.1002/mco2.70080

**Published:** 2025-02-21

**Authors:** Lingfeng Bi, Xin Wang, Jiayi Li, Weimin Li, Zhoufeng Wang

**Affiliations:** ^1^ Department of Respiratory and Critical Care Medicine, Frontiers Science Center for Disease‐related Molecular Network, State Key Laboratory of Respiratory Health and Multimorbidity West China Hospital, Sichuan University Chengdu Sichuan China; ^2^ Institute of Respiratory Health, Frontiers Science Center for Disease‐Related Molecular Network West China Hospital, Sichuan University Chengdu Sichuan China; ^3^ Precision Medicine Center, Precision Medicine Key Laboratory of Sichuan Province West China Hospital, Sichuan University Chengdu Sichuan China; ^4^ The Research Units of West China, Chinese Academy of Medical Sciences West China Hospital Chengdu Sichuan China

**Keywords:** DNA methylation, epigenetics, histone modification, lung cancer, noncoding RNA regulation

## Abstract

The integration of liquid biopsy with epigenetic markers offers significant potential for early lung cancer detection and personalized treatment. Epigenetic alterations, including DNA methylation, histone modifications, and noncoding RNA changes, often precede genetic mutations and are critical in cancer progression. In this study, we explore how liquid biopsy, combined with epigenetic markers, can provide early detection of lung cancer, potentially predicting onset up to 4 years before clinical diagnosis. We discuss the challenges of targeting epigenetic regulators, which could disrupt cellular balance if overexploited, and the need for maintaining key gene expressions in therapeutic applications. This review highlights the promise and challenges of using liquid biopsy and epigenetic markers for early‐stage lung cancer diagnosis, with a focus on optimizing treatment strategies for personalized and precision medicine.

## Graphical abstract

Epigenetic changes like DNA methylation and histone modifications, key from atypical adenomatous hyperplasia to stage I lung cancer, precede genetic mutations and contribute to tissue dysplasia. Along with mRNA, noncoding RNA, and tumor microenvironment regulation, these markers, enhanced by liquid biopsy, are promising for early lung cancer diagnosis.

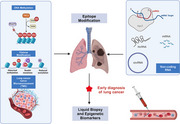

## 1 INTRODUCTION

Lung cancer, a leading cause of cancer‐related mortality worldwide, poses a significant threat to public health. According to National Cancer Report 2024, lung cancer is responsible for around 1.1 million new cases and 0.74 million deaths in 2022,^1^ highlighting its devastating impact.^2^ Lung cancer is divided into small cell lung cancer (SCLC) and non‐small cell lung cancer (NSCLC). NSCLC includes squamous cell carcinoma, adenocarcinoma, and large cell carcinoma. Among these, NSCLC accounts for 80–85% of cases. However, compared with NSCLC, SCLC, characterized by small cell size, rapid growth, and early metastasis, typically has a poorer prognosis with a lower 5‐year survival rate.^3^ The key to effective treatment and improving survival rates lies in early detection. Yet, the early stages of lung cancer often go unnoticed due to a lack of understanding about its initial progression, with more than 80% of cases diagnosed at an advanced stage.^4^ This is one of the reasons why lung cancer continues to have such a high mortality rate. Therefore, it is crucial to intensify research into the early mechanisms of lung cancer and develop effective screening and diagnostic methods to lower mortality rates.^5^ Recently, the focus has shifted to the role of epigenetics in the early stages of the disease. As research into early‐stage lung cancer progresses, it has been discovered that in addition to genetic mutations, epigenetic changes are also closely associated with the development of NSCLC and may even become the main factor in early‐stage lung cancer.^6^ Because epigenetic changes occur earlier than genetic mutations, they hold significant potential as biomarkers for the diagnosis of early‐stage lung cancer.^7^ Although epigenetic modifications are common in lung cancer, their impact on the early progression of the disease is still not well understood, making this an area of keen scientific interest.

Although the treatment and diagnostic techniques for early‐stage lung cancer have evolved from simple chest X‐rays in the early 20th century to advanced computed tomography (CT) scans, positron emission tomography (PET) scans, and MRI technologies, these methods still have limitations in sensitivity and specificity for early‐stage lung cancer.^8^ Despite the introduction CT by Godfrey Hounsfield in the 1960s and the subsequent development of PET scans and MRI, which improved resolution of lung structures,^9^ these technologies are still limited in detecting small tumors and precancerous changes, and they are costly and complex to operate. For example, although low‐dose CT has improved the detection of early‐stage lung cancer in screening, it still poses risks of overdiagnosis and unnecessary interventions. Entering the 21st century, the development of molecular biology techniques has made liquid biopsy and genetic sequencing important diagnostic tools supporting personalized treatment.^10^ Liquid biopsy is a noninvasive diagnostic method that detects cancer by analyzing tumor DNA and other biomarkers in the blood. In combination with epigenetic markers, especially DNA methylation, liquid biopsy has shown significant potential in the detection of early stage lung cancer. Epigenetic modifications such as DNA methylation and histone modification occur very early in the development of lung cancer, often preceding traditional genetic mutations, providing new biomarkers for early diagnosis.^11^ The DNA methylation patterns of patients with NSCLC can serve as effective markers for early diagnosis and prognosis assessment.^12^ Detection of epigenetic markers can noninvasively reveal the presence of lung cancer and provide critical information about the potential development pathways and treatment responsiveness of tumors,^13^ which is significant for designing more effective personalized treatment strategies. Moreover, early‐stage lung cancers have a lower frequency of genetic mutations while epigenetic modifications appear earlier, potentially serving as the primary cause of early‐stage lung cancer and promising molecular markers for early diagnosis, promoting the development of early diagnostic methods.^11^ Additionally, reversing abnormal epigenetic changes has become a potential strategy for treating cancer. Many compounds targeting enzymes regulating histone acetylation, histone methylation, and DNA methylation have been developed as epigenetic therapies, some of which have shown efficacy in hematologic malignancies and solid tumors.^14^

**FIGURE 1:**
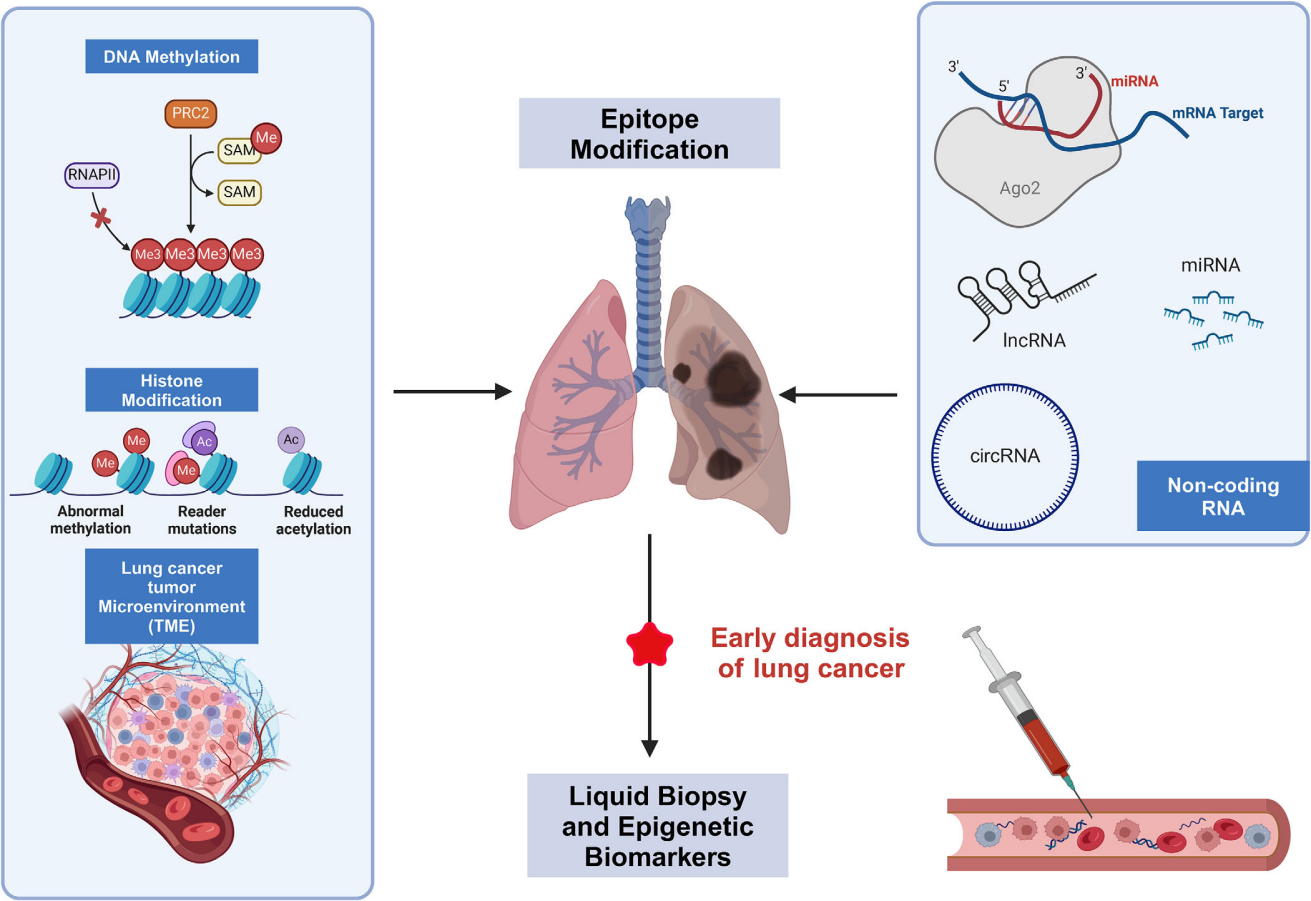
Factors contributing to the development of early stage lung cancer (DNA methylation, histone modifications, mRNA, noncoding RNA, and tumor microenvironment [TME] regulation). Epigenetic modifications, such as DNA methylation and histone modification, occur in the early stages of lung cancer, from atypical adenomatous hyperplasia to stage I, and are associated with tissue dysplasia. These changes typically precede characteristic genetic mutations. When combined with liquid biopsy techniques, these epigenetic molecular markers hold potential as critical biomarkers for the early diagnosis of lung cancer. Created with BioRender.com

**FIGURE 2:**
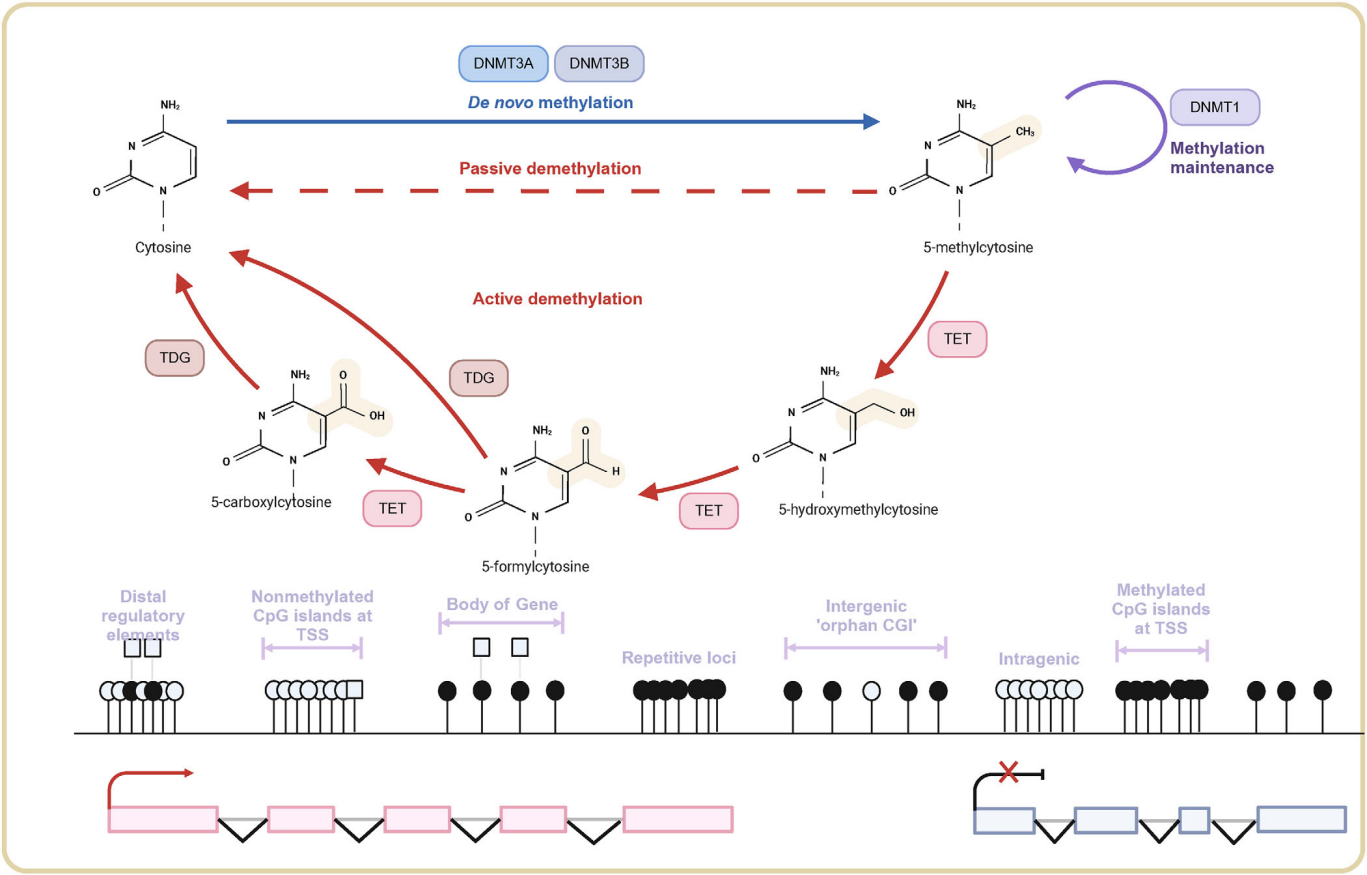
Establishment and regulation of methylation patterns in lung cancer. Methylation modifies different genomic environments such as promoters, gene bodies, and intergenic regions either by directly blocking the binding of transcription factors or by altering chromatin structure, methylation regulates the accessibility of genes, which in turn affects gene expression. Enzymes such as DNMT3A and DNMT3B are responsible for methylation from the head of the DNA, which is the addition of methyl groups to previously unmethylated DNA. In contrast, DNMT1 maintains methylation patterns during DNA replication, ensuring that the DNA methylation state is passed on to daughter cells after cell division. The lower half of the figure shows the process of active and passive DNA demethylation. Passive demethylation occurs when there is a lack of methylation maintenance during DNA replication, which results in the gradual loss of methylation marks. Active demethylation, on the other hand, is a more dynamic, enzyme‐mediated process that is accomplished primarily by TET enzymes. These enzymes progressively remove 5‐methylcytosine (5mC) through a series of oxidative steps, and thymine DNA glycosylase (TDG), which is essential for base excision and substitution, returns to the unmethylated state after this step. For regulatory elements such as enhancers that are located distally, the methylation state can also affect gene expression at some distance. In the case of lung cancer, this regulation may lead to the silencing of tumor suppressor genes or the activation of oncogenes. Created with BioRender.com.

This review discusses the promising application of combining liquid biopsy with epigenetic markers in the early diagnosis of lung cancer, showcasing significant developmental potential. Researchers have focused for decades on epigenetic modifications during the DNA templating process, including DNA methylation, histone modifications, noncoding RNA (ncRNA), mRNA, and epigenetic changes within the tumor microenvironment (TME), all of which play critical roles in cancer progression. Notably, epigenetic alterations within the TME not only impact tumor immunogenicity but also regulate immune cells involved in antitumor responses. Current clinical data indicate that liquid biopsy detection of DNA methylation can predict cancer occurrence up to 4 years in advance, demonstrating that these epigenetic alterations usually precede common genetic mutations and are effective therapeutic targets. Moreover, although epigenetic regulators may need to maintain the expression of certain key target genes in tumor therapy, broadly targeting these factors could disrupt cellular balance, leading to cellular catastrophe. Therefore, this review not only explores the prospects of using liquid biopsy and epigenetic markers in early lung cancer diagnosis and treatment but also highlights the main challenges faced by this approach, emphasizing the necessity for further research to optimize treatment strategies and ultimately achieve personalized medicine and precision therapy (Figures 1, 2).

## 2 DEVELOPMENT OF EARLY‐STAGE LUNG CANCER

The progression of lung cancer from atypical adenomatous hyperplasia (AAH) to minimally invasive stage IA is a critical stage in the early development of lung cancer,^15^ involving multilevel biological changes and interactions among molecular signaling pathways. AAH, as a potential precursor to lung cancer, is characterized by mild to moderate cellular atypia and these cells are primarily confined to the alveolar walls.^16^ At this early stage, the activation of the Wnt/β‐catenin pathway is crucial, as it promotes the accumulation of β‐catenin in the nucleus and activates the expression of genes such as cyclin D1 and c‐myc, thereby driving the cell cycle progression and cell proliferation.^17^ As the lesion progresses to adenocarcinoma in situ (AIS) and minimally invasive adenocarcinoma, mutations or overexpression in the epidermal growth factor receptor (EGFR) pathway also begin to show their significance.^18^, ^19^ These changes lead to enhanced cell proliferation and survival signals, further promoting tumor growth and progression. Additionally, epigenetic regulatory mechanisms, especially changes in DNA methylation and histone modifications, start to play a more prominent role at this stage.^20^, ^21^ High levels of DNA methylation may lead to the silencing of key tumor suppressor genes,^22^ while changes in histone acetylation and methylation affect the structure of chromatin and gene expression.^23^ The TME, including the extracellular matrix around the tumor, immune cells, and tumor‐associated fibroblasts, provides necessary support and protection for the tumor.^24^ Remodeling of the extracellular matrix through the regulation of matrix metalloproteinases (MMPs) provides a physical pathway for the invasion and spread of tumor cells.^25^ Moreover, immune cells such as tumor‐associated macrophages contribute to a tumor‐promoting state in the TME by secreting proinflammatory and progrowth factors.^26^

Therefore, a deeper understanding of the specific roles and regulatory mechanisms of the Wnt signaling pathway in early‐stage lung cancer, especially at the molecular and epigenetic levels, is crucial for revealing the cellular and molecular mechanisms of early lung cancer development. This knowledge also provides important molecular targets for the development of targeted therapies aimed at this pathway, potentially improving treatment strategies and enhancing the survival rates of patients with early‐stage lung cancer.

### 2.1 Molecular and cellular changes in early lung cancer

The formation of lung cancer fundamentally represents a complex transition from normal cells to cancerous cells. In the development of early‐stage lung cancer, the transformation from pre‐malignant lesions to carcinoma is critically decisive. Specifically, during the progression from AAH to AIS and minimally invasive adenocarcinoma, numerous molecular markers exhibit significant changes.^27^ These markers include key components of the Wnt/β‐catenin and EGFR signaling pathways,^28^ whose activation propels the entire process from cell cycle progression to cell proliferation.^15^ The activation of these signaling pathways not only promotes cellular growth but also enhances the tumor cells capacity to resist apoptosis, thereby further driving tumor growth and progression.

In different subtypes of lung cancer, these molecular alterations confer distinct genetic and phenotypic characteristics. For instance, lung adenocarcinoma (LUAD) often demonstrates mutations or overexpression of EGFR,^29^ while lung squamous cell carcinoma may exhibit alterations in gene expression related to keratins.^30^ SCLC is characterized by frequent inactivation of genes associated with neuroendocrine differentiation, such as RB1 and TP53.^31^ These features suggest that the different subtypes of lung cancer may originate from various types of lung stem or progenitor cells, each following a distinct developmental trajectory and exhibiting different therapeutic responses.

Furthermore, the application of large‐scale parallel sequencing technologies has enabled the precise identification of genetic mutations closely associated with the development of lung cancer. These genetic discoveries not only provide new insights into the mechanisms of lung cancer formation but also propel the development of therapeutic approaches targeting specific molecular markers. For example, targeted therapies against EGFR mutations have become a crucial component of NSCLC treatment, significantly improving the survival rates of specific patient groups. This strategy of treatment based on molecular markers offers more personalized and effective options for early‐stage lung cancer, potentially enhancing the prognosis and quality of life for patients with lung cancer.

### 2.2 Role of epigenetics in early tumorigenesis

In the field of oncology, significant progress has been made in epigenetic research. Epigenetic modifications play an important role in the development of lung cancer. The identification of epigenetic diagnostic and prognostic biomarkers as well as epigenetic‐based therapeutic approaches are becoming hot research topics.

DNA methylation plays a crucial role in suppressing gene activity and maintaining genomic stability by inhibiting recombination events between repetitive sequences.^32^ The silencing of inhibitors or the increased expression of activators caused by DNA methylation is a common mechanism for abnormal activation of the Wnt pathway.^33^, ^34^ Given the regulatory effects of DNA methylation on the Wnt signaling pathway, drugs targeting DNA methylation (such as DNMT inhibitors) and those targeting the Wnt pathway are effective in inhibiting tumor progression. Histone modification is another important aspect of epigenetic regulation, affecting the expression of oncogenes through mechanisms such as acetylation, deacetylation, and methylation, thereby leading to lung cancer.^35^, ^36^ Acetylation modifications promote gene expression by opening chromatin structures, allowing transcription factors to more easily bind to promoter regions, which sustains activation of the Wnt pathway in lung cancer cells, further promoting tumor proliferation and aggressiveness.^37^, ^38^ Due to the significance of histone modifications in regulating the Wnt signaling pathway, drugs targeting histone modification enzymes including HDACs, HATs, and HMTs are considered to have potential therapeutic value.^39^, ^40^ In the development of early‐stage lung cancer, ncRNA plays a critical regulatory role by finely tuning the expression and activity of specific factors within key signaling pathways. These ncRNAs, including microRNA (miRNA), long ncRNA (lncRNA), and circular RNA (circRNA), significantly regulate tumor behavior and its microenvironment through multiple pathways affecting cell proliferation, migration, invasion, and apoptosis.^41^, ^42^ In terms of drug development, research on ncRNA has shown potential therapeutic prospects. For instance, miRNA‐based treatment strategies, including the development of synthetic miRNA mimics or anti‐miRNA oligonucleotides, are currently being developed to restore or suppress the function of specific ncRNAs, regulating key pathways in lung cancer.^43^, ^44^ These therapies have demonstrated potential in preclinical studies and early clinical trials, especially for patients with specific ncRNA expression abnormalities.^45^, ^46^ The expression patterns and regulatory mechanisms of mRNA are crucial for understanding the pathogenesis of tumors. In particular, the roles of mRNA in regulating the Wnt/β‐catenin and PI3K/Akt/mTOR pathways are significant.^47^, ^48^ Overexpression of β‐catenin mRNA observed in many early‐stage lung cancer samples leads to sustained activation of the Wnt signaling pathway, promoting cell proliferation and inhibiting apoptosis, thereby supporting tumor development and spread.^49^, ^50^ Its widespread use in targeted therapy, the detection and analysis of mRNA expression have become important research tools.^51^ The TME plays a critical role in the progression of early‐stage lung cancer, particularly the roles of macrophages, exosomes, and T cells in regulating tumor behavior and the interactions between lung cancer cells and surrounding cells.^52^, ^53^ Exosomes released by lung cancer cells are rich in miRNAs that can activate or inhibit the Wnt signaling pathway, thereby affecting lung cancer cell behavior and microenvironment regulation.^54^ The close connection between ncRNA and the TME allows targeting specific miRNAs and lncRNAs to significantly influence the behavior of macrophages and T cells in the TME,^55^ thereby regulating the development of lung cancer. Therefore, utilizing epigenetic modification factors in the TME as biomarkers for detecting early‐stage lung cancer has become a current research focus.

## 3 LIQUID BIOPSY AND EPIGENETIC BIOMARKERS IN EARLY LUNG CANCER DIAGNOSIS

### 3.1 Liquid biopsy techniques

Liquid biopsy, valued for its simplicity, speed, noninvasiveness, repeatability, and real‐time monitoring capabilities, is widely regarded as a potent tool for the future of early lung cancer screening and diagnosis.^56^ This technique identifies tumor occurrence and progression information by analyzing tumor cells or nucleic acids in bodily fluids, including blood, urine, cerebrospinal fluid, saliva, bronchoalveolar lavage fluid, and pleural effusion.^57^

The integration of epigenetic biomarkers, such as DNA methylation, has shown significant promise in enhancing the prospects of early lung cancer diagnosis. Liquid biopsy utilizes components like circulating tumor DNA (ctDNA), circulating tumor cells (CTCs), and exosomes from bodily fluids to detect genetic and epigenetic alterations specific to tumors.^58^, ^59^ These alterations, especially patterns of gene methylation which occur in the promoter regions of tumor suppressor genes or oncogenes, provide highly sensitive and specific methods for early diagnosis. For instance, studies have demonstrated that the combined methylation analysis of SHOX2 and RASSF1A in bronchoalveolar lavage fluid significantly enhances overall diagnostic accuracy compared with traditional cytological examination and serum biomarkers like carcinoembryonic antigen.^60^ Moreover, the relative stability of DNA methylation allows for its detection in noninvasive samples, offering a more precise approach for early screening and diagnosis.

Another crucial component of liquid biopsies is exosomes, small lipid bilayer vesicles generated by cells through an endocytosis–fusion–exocytosis mechanism, measuring approximately 30–100 nm in diameter.^61^ These vesicles, which are more abundant in the bodily fluids of lung cancer patients compared with healthy individuals, serve as carriers for intercellular material and information exchange.^62^ Exosomes maintain intracellular homeostasis and transfer DNA, mRNA, ncRNAs, and proteins. These molecules play pivotal roles in promoting TME formation, enhancing tumor invasiveness, mediating immune escape, and inducing therapeutic resistance.^63^ Thus, investigating the potential mechanisms of exosomes in tumor development could provide new insights for early diagnosis, prognosis, and resistance evaluation of lung cancer.

With the rapid development of genomics, transcriptomics, proteomics, and other multiomics integrations, alongside the application of high‐throughput sequencing technologies, liquid biopsy is poised to delve deeper into biological information, offering more accurate early lung cancer diagnoses and tailored treatment plans. By leveraging these advanced technologies, liquid biopsy not only improves early detection and prognostic assessment of lung cancer but also contributes to the realization of precision medicine, ultimately enhancing survival rates and quality of life for lung cancer patients.

### 3.2 Epigenetic biomarkers

#### 3.2.1 DNA methylation

DNA methylation, a key epigenetic modification, primarily occurs in the cytosine of CG dinucleotides, forming 5‐methylcytosine. Catalyzed by DNA methyltransferase using S‐adenosylmethionine, it leads to methylation of up to 90% of CpGs in the genome, with unmethylated CpGs clustering in CpG islands (CGIs) at gene promoters and transcription start sites.^64^, ^65^ Lung cancer often features aberrant DNA methylation, including both hypo‐ and hypermethylation, impacting gene expression and contributing to cancer progression.^66^, ^67^

DNA methylation plays a crucial role in suppressing gene activity and maintaining genomic stability by inhibiting recombination events between repetitive sequences. The silencing of inhibitors or the increased expression of activators caused by DNA methylation is one of the common mechanisms for the abnormal activation of the Wnt pathway.^68^ For example, the promoter methylation of genes such as those in the DKK family (e.g., DKK1, DKK3), the SFRP family (e.g., SFRP1, SFRP2),^69^, ^70^ and WIF1 (Wnt Inhibitory Factor 1) can lead to their downregulation or silencing, thereby lifting the inhibition on the Wnt pathway and causing its abnormal activation, which in turn promotes the proliferation and survival of tumor cells.^71^, ^72^ In some cases, positive regulators in the Wnt signaling pathway may be activated due to hypomethylation or other mechanisms. Increased expression and activity of β‐catenin lead to the sustained activation of the Wnt signaling pathway, promoting the proliferation, invasion, and metastasis of tumor cells.^73^ Given the regulatory effects of DNA methylation on the Wnt signaling pathway, drugs targeting DNA methylation (such as DNMT inhibitors) and those targeting the Wnt pathway may be effective in inhibiting tumor progression. Research is also exploring how to reverse these methylation changes to restore normal regulation of the Wnt signaling pathway (Figure 2).

##### Hypomethylation

Like many cancers, lung tumors are particularly prone to widespread hypomethylation, especially targeting repetitive sequences such as SINEs, LINEs, subtelomeric repeats, and segmental duplications.^74^, ^75^ This reduction in methylation at LINE‐1 is linked with poorer outcomes and is observed even in the absence of mutations in key driver genes of LUAD.^76^, ^77^ While hypomethylation at transcriptional regulatory elements is less common than hypermethylation, it nonetheless has the potential to activate oncogenes. For instance, the demethylation of CGIs within the SNCG gene correlates with its heightened expression observed in various solid tumors, including lung cancer.^78^ Studies also indicate that SNCG is involved in the migration and invasion of cancer cells.^79^ Similarly, research shows that MAGE genes, upregulated in 41.8–46.3% of NSCLC tumors and carcinogen‐affected lung epithelial cells, are also associated with a loss of methylation.^80^ Overexpression of these genes is crucial for tumor growth and metastasis and is associated with poor prognosis in lung cancer.^81^ Moreover, hypomethylation in tumors may trigger genomic instability by reactivating retrotransposon elements or inducing chromosome rearrangements. For example, hypomethylation at the 3′tandem repeat region of the HRAS gene in lung cancer could lead to gene loss.^82^ An increase in the hypomethylation of retrotransposable elements (LINE‐1 and Alu) is strongly linked with enhanced transcription and increased genomic instability in lung cancer.^83^ This is supported by findings that show a correlation between increased global hypomethylation and higher rates of mutation, copy number variations, and allelic imbalances, as well as shifts in the Treg/CD8 ratio during lung cancer progression (as summarized in Table 1).

**TABLE 1:** Select genes reported to undergo promoter methylation frequently in lung cancer.

Gene	Locus (Ensembl)	Frequency in lung cancer (%)	Gene function	Hyper/hypomethylation	References
HOXA4	7p15.2	20	Play a role in inhibiting the growth, invasion, and migration of lung cancer cells. Overexpressing inhibited cell growth and promoted apoptosis, while silencing it had the opposite effect and led to a decrease in lung cancer cell migration and invasion.	Hypermethylation	^112^, ^113^
TSLC1	11q23.3	37–44	It is involved in suppressing the malignant phenotype of cancer cells. Can alter the growth properties of lung cancer cells. Modify the expression of various genes involved in important cellular processes such as cell cycle regulation, signaling pathways, and metastasis.	Hypermethylation	^114^, ^115^, ^116^, ^117^, ^118^, ^119^
SHOX2	3q25.32	91–95	Homeobox gene family is implicated in gene transcription and is potentially involved in regulating cell growth and differentiation.	Hypermethylation	^120^, ^121^, ^122^, ^123^
RUNX3	1q36.11	25	A transcription factor containing the Runt domain functions as a tumor suppressor gene.	Hypermethylation	^124^, ^125^, ^126^, ^127^, ^128^, ^129^
DAPK	9p21.33	40.5–40.6	Associated with tumor metastasis and poor prognosis in lung cancer. And as a tumor suppressor whose inactivation may contribute to the progression of lung cancer	Hypermethylation	^90^, ^91^, ^130^, ^131^, ^132^
CDH1	16q22.1	12–58	Associated with tumor invasiveness, metastasis, and poor prognosis; Decreased expression of CDH1 can facilitate cancer cells to detach from the primary tumor, spreading through vascular or lymphatic systems, leading to cancer dissemination	Hypermethylation	^106^, ^133^, ^134^, ^135^, ^136^
CDH13	16q23.3	28–30	Tumor suppressor gene, is frequently hypermethylated and silenced in lung cancer, contributing to tumor progression and metastasis, and serving as a potential biomarker for diagnosis and prognosis	Hypermethylation	^137^, ^138^, ^139^
CDKN2A/P16	9p21.3	22–47	Acting as a tumor suppressor by inhibiting cyclin‐dependent kinases, is frequently inactivated in lung cancer, leading to unchecked cell proliferation and contributing to cancer progression.	Hypermethylation	^140^, ^141^
FHIT	3p14.2	34–47	Functioning as a tumor suppressor, is often deleted or underexpressed in lung cancer, contributing to increased tumorigenesis and a potential marker for poor prognosis.	Hypermethylation	^142^, ^143^, ^144^
GSTP1	11q13.2	33.7	Primarily involved in detoxification processes, exhibits altered expression in lung cancer, influencing drug resistance and potentially impacting the effectiveness of chemotherapy	Hypermethylation	^145^, ^146^, ^147^
APC	5q22.2	30–96	Tumor suppressor gene, is often mutated or downregulated in lung cancer, leading to aberrant Wnt signaling and contributing to tumorigenesis and progression.	Hypermethylation	^102^, ^148^
RASSF1A	3p21.31	25–45	Tumor suppressor gene, is frequently inactivated in lung cancer through hypermethylation, contributing to tumor progression and serving as a potential biomarker for early detection and prognosis.	Hypermethylation	^149^, ^150^, ^151^, ^152^
MGMT	10q26.3	11–38	Plays a crucial role in repairing DNA damage in lung cancer, and its expression levels are associated with resistance to alkylating agents, impacting treatment efficacy	Hypermethylation	^96^, ^153^, ^154^
MAGE	Xq28	41.8–46.3	Transcriptional regulation, cancer development, and progression	Hypomethylation	^155^, ^156^, ^157^
SNCG	10q23.2	Not clear	Cell migration and invasion	Hypomethylation	^79^

These insights highlight the multifaceted role of DNA hypomethylation in fostering chromosomal instability, driving mutagenesis, and altering the TME. Although global hypomethylation is often seen in tumor cells, it can also correlate with compact, relatively inactive genomic regions.^84^ This reorganization can trigger antitumor immune responses and suppress genes involved in stemness, invasion, and metastasis across various cancers, suggesting that the prevalent global hypomethylation in tumors might result from cumulative cell divisions, potentially playing a tumor‐suppressive role rather than an oncogenic one.

##### Hypermethylation

While research on hypomethylation is not as extensive, there is a wealth of studies focusing on how hypermethylation affects lung cancers. The inconsistencies in research findings often arise from differences in study designs, types of samples used (like primary tumors versus cell lines), the various histological types of lung cancer, and the methods employed (ranging from quantitative to qualitative analyses, varying numbers of CpG sites studied, different microarray technologies, and diverse statistical approaches).^85^ Notably, many potential tumor suppressor genes have been found to be consistently hypermethylated in lung cancers.^86^, ^87^, ^88^ These genes are pivotal in managing key cellular functions that frequently become disrupted in cancer, such as apoptosis (involving genes like CASP8 and DAPK),^89^, ^90^, ^91^, ^92^ cell cycle control (e.g., CDKN2A/p16 and PTEN),^38^, ^93^, ^94^, ^95^, ^96^, ^97^, ^98^ DNA repair (MGMT, MLH1),^99^, ^100^ and pathways regulating signaling and cell adhesion (like APC and CDH1).^101^, ^102^, ^103^, ^104^, ^105^, ^106^, ^107^ Treatment with methylation inhibitors such as 5‐aza‐2′ deoxycytidine in lung cancer cell lines has been shown to reactivate many of these suppressed genes,^108^ confirming their regulation through DNA methylation. Though methylation typically leads to gene silencing, it has also proven useful for identifying epigenetic markers in lung cancer.^109^

Intriguingly, some genes exhibit both hypermethylation and hypomethylation, suggesting a sophisticated regulatory mechanism that affects gene expression in complex ways. For example, the UNC5D gene shows hypermethylation at its promoter and hypomethylation in downstream introns in lung cancer.^110^ Moreover, hypermethylation doesn't always suppress genes; it can activate them. A notable instance is the hypermethylation of the TERT promoter, which has been found to increase TERT expression in most tumor types, including lung cancers.^104^ While similar disruptions by hypermethylation have been observed in other cancers, their effects in lung cancers are still being explored^111^ (as summarized in Table 1).

These findings emphasize the need to understand the specific epigenetic changes linked to different lung cancer subtypes, smoking status, and genetic alterations. Such detailed knowledge could help uncover distinct mechanisms, assist in the development of targeted diagnostic and prognostic markers, and guide personalized treatment strategies.

DNA methylation studies are important in the detection and treatment of lung cancer. These studies focus on the potential of methylation as a molecular marker and its application in early cancer detection, chemoprevention, diagnosis, treatment or prognosis. Methylation changes usually occur at CGIs in the promoter regions of genes, which makes them ideal biomarkers that can be easily detected by methods such as PCR. In addition, by analyzing the DNA methylation status of a large number of loci, it helps to identify molecular differences between tumors and normal tissues, as well as between different tumor subtypes. This information is expected to be used for the discovery of new markers to improve the detection and diagnoses plan of early‐stage lung cancer.^158^

#### 3.2.2 Histone modification

**FIGURE 3:**
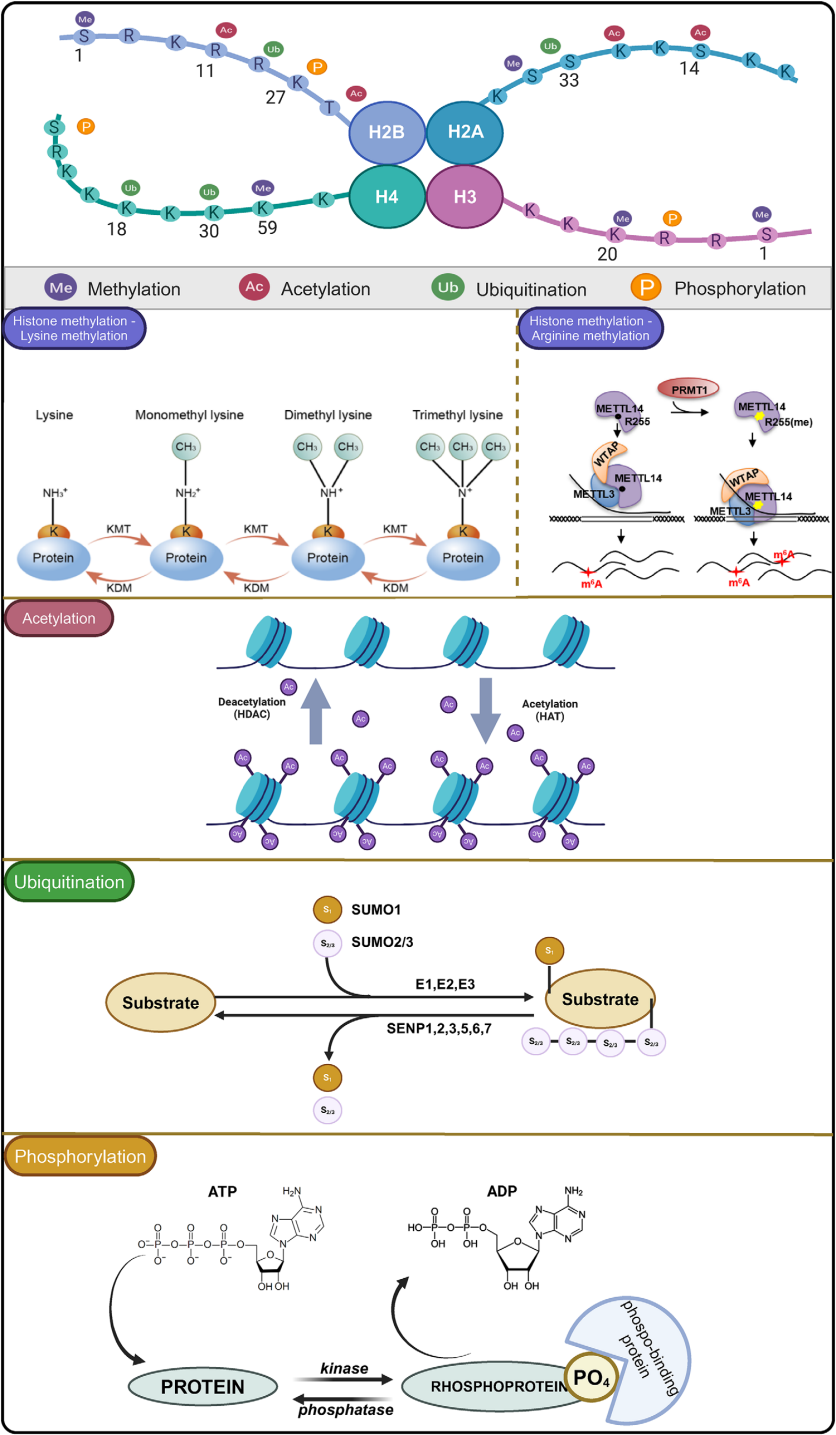
Histone modification mechanisms (including methylation, acetylation, ubiquitination and phosphorylation) are common in lung cancer. Histone methylation occurs through mono‐, di‐, or trimethylation on histones H3 and H4 by lysine methyltransferases (KMTs) and is then reversed by lysine demethylases (KDMs). Histone arginine methylation is catalyzed by protein arginine methyltransferases (PRMTs) and plays an important role in transcriptional regulation of chromatin structure. Histone acetylation occurs through the addition of acetyl groups to specific lysine residues by histone acetyltransferases (HATs), and acetylation leads to the opening of chromatin structures, which enables active gene transcription. Histone deacetylation, on the other hand, removes these acetyl groups by histone deacetylases (HDACs), resulting in a tight chromatin structure and reduced gene expression. The balance between acetylation and deacetylation plays a crucial role in regulating gene expression patterns in tumor cells. In addition to this, ubiquitination regulates histones by adding ubiquitin proteins, whereas phosphorylation affects cellular processes by adding phosphate groups to specific residues. In contrast, phosphatases are able to reverse phosphorylation modifications and maintain homeostasis within the cell. These modifications described above do not exist in isolation, but are part of an interaction that affects chromatin accessibility, recruitment of transcription factors, and regulation of gene expression. Created with BioRender.com.

Histone modifications play a pivotal role in the onset and progression of lung cancer, significantly influencing tumor cell function through the fine‐tuning of chromatin structure and gene expression. These modifications, including but not limited to acetylation, methylation, and phosphorylation, alter the interaction between histones and DNA, thereby regulating gene accessibility and expression (Figure 3).

Mechanisms of histone modification include methylation, where lysine residues of histones can be methylated to form different methylation states.^159^, ^160^ This affects chromatin structure and gene accessibility.^161^ Phosphorylation, that is, phosphorylation modifications can regulate the interaction of histones with other proteins and affect gene expression.^162^ Ubiquitination, that is, ubiquitination modifications are involved in protein degradation and signaling. It also includes crotonylation, glycosylation, and ADP‐ribosylation. Histone modification imbalance is closely related to tumorigenesis and development.^163^ In particular, the methylation and acetylation status of histone H3 and H4 residues have been shown to be markers of tumor cells. Acetylation, that is, acetylation modifications are usually associated with gene activation, relaxing chromatin and promoting gene transcription.^164^ Acetylation modifications open chromatin structures, making it easier for transcription factors to bind to promoter regions, thereby promoting gene expression. This mechanism leads to the sustained activation of the Wnt pathway in lung cancer cells, which in turn promotes tumor proliferation and aggressiveness.^165^ Histone acetylation is often associated with gene activation; when histones around positive regulatory genes in the Wnt signaling pathway (such as those encoding β‐catenin by CTNNB1) undergo acetylation, the transcriptional activity of these genes increases, leading to the activation of the Wnt pathway. Histone deacetylation usually results in gene silencing; reduced transcriptional activity of Wnt pathway inhibitory genes, such as those in the DKK and SFRP families, may be due to histone deacetylation, which can lead to decreased expression of these inhibitors, thereby lifting the inhibition on the Wnt pathway.^166^ At the same time, histone deacetylases (HDACs) play a key role in this process. HDAC inhibitors like Vorinostat, by blocking deacetylation, may reactivate these inhibitory genes and counteract the abnormal activation of the Wnt signa.^167^ Methylation also plays a role in histone modification; if H3K9 methylation occurs in the promoter regions of Wnt pathway inhibitory genes, these genes may be silenced, leading to the overactivation of the Wnt signaling pathway. Conversely, increased H3K4 methylation may promote the expression of Wnt pathway activation genes, further driving the progression of lung cancer.^168^ Due to the importance of histone modifications in regulating the Wnt signaling pathway, drugs targeting histone modifying enzymes including HDACs, HATs, and HMTs are considered to have potential therapeutic value. For example, HDAC inhibitors can partially reverse the abnormal activation of the Wnt signaling pathway by restoring the expression of Wnt inhibitory factors, thereby inhibiting the progression of lung cancer.^169^

In lung cancer, aberrant patterns of histone modifications can lead to the downregulation of key tumor suppressor genes or the overexpression of oncogenes, thus promoting tumor growth and dissemination. Specific abnormalities in histone modification, such as the trimethylation of histone H3 on lysine 27 (H3K27me3), have been identified to increase in numerous cases of lung cancer. This modification suppresses the expression of a cohort of tumor suppressor genes, facilitating the proliferation and invasion of lung cancer cells. Moreover, histone modifications affect not only the expression of individual genes but also entire gene expression networks and signaling pathways, including cell cycle regulation, DNA repair, apoptosis, and intercellular signaling, further driving the advancement of lung cancer. YEATS2, a YEATS domain‐containing protein, has been increasingly recognized for its critical role in the pathogenesis of lung cancer.^170^ Research has shown that YEATS2 is highly amplified in human lung cancer and is essential for cancer cell growth and survival.^171^ The protein binds to acetylated histone H3 through its YEATS domain, influencing gene expression and chromatin structure in cancer cells. This interaction significantly alters the transcriptional program, which is essential for lung cancer tumorigenesis.YEATS2 is not only amplified in lung cancer but also required for the growth and survival of cancer cells.^172^, ^173^ The research highlighted that the YEATS2‐containing ATAC complex colocalizes with H3K27 acetylation on the promoters of actively transcribed genes.^174^ The depletion of YEATS2 or disruption of its interaction with acetylated histones reduces promoter H3K9ac levels and deactivates the expression of essential genes, underlying its pivotal role in lung cancer tumorigenesis (YEATS2 links histone acetylation to tumorigenesis of NSCLC).Furthermore, the role of YEATS2 in various tumors, overexpression of YEATS2 promotes the proliferation and migration of pancreatic cancer cells, and it has been associated with the progression of lung cancer through histone acetylation. YEATS2 also can act as a target of HIF1α and regulate the TAK1/NF‐κB pathway to promote tumor progression in pancreatic cancer, highlighting its potential as a therapeutic target in various cancer types including lung cancer (YEATS2 is a target of HIF1α and promotes pancreatic cancer cell proliferation and migration).These findings underscore the significance of YEATS2 in the molecular pathology of lung cancer, suggesting its potential as a target for therapeutic intervention in cancer treatment.

#### 3.2.3 Messenger RNA modification

The expression patterns and regulatory mechanisms of mRNA play a pivotal role in tumor biology, impacting not only the functionality of genes but also profoundly influencing the pathogenesis of tumors. The regulation of mRNA is particularly critical across many essential signaling pathways, especially concerning tumor progression and the development of treatment resistance. For instance, in the Wnt/β‐catenin pathway, a direct correlation exists between the abnormal activation in lung cancer and the expression levels of β‐catenin mRNA.^175^, ^176^ In many early‐stage lung cancer specimens, a significant overexpression of β‐catenin mRNA has been observed.^177^, ^178^ This upregulation not only triggers continuous activation of the Wnt signaling pathway but also propels the cell cycle forward, enhancing cellular proliferation while inhibiting apoptosis, collectively fostering tumor growth and metastasis.^179^, ^180^

Moreover, the PI3K/Akt/mTOR signaling pathway serves as a critical regulatory axis in tumor development.^181^, ^182^ Within this pathway, the regulation of Akt mRNA levels is crucial for the progression of lung cancer. Aberrant expression of Akt mRNA often leads to excessive activation of Akt protein, enhancing tumor cell survival and proliferation capabilities and potentially increasing resistance to various therapeutic approaches.^183^, ^184^ Consequently, the expression and functional status of Akt mRNA and protein have become significant targets in lung cancer treatment. In modern oncology, the detection and analysis of mRNA expression are instrumental in identifying tumor biomarkers.^185^, ^186^

In addition to coding RNAs, the role of ncRNAs (such as miRNAs and lncRNAs) in lung cancer is increasingly being elucidated. Recent advances in therapeutics based on miRNA and lncRNA are actively being pursued. These strategies, by targeting and modulating specific ncRNAs, can precisely regulate their roles in critical signaling pathways of lung cancer, thus offering new therapeutic options.^187^ For instance, by restoring or inhibiting specific miRNAs, it is possible to adjust tumor cell behaviors, affecting their proliferation, migration, or apoptosis, providing more precise molecular targeting for the treatment of early‐stage lung cancer. These studies not only enrich our understanding of the molecular mechanisms of lung cancer but also open new avenues and strategies for future cancer therapies.

#### 3.2.4 ncRNA regulation

In the human genome, genes with the function of coding proteins account for only 2% of all genomic sequences, and the majority of the remaining 98% of the sequences are transcribed into ncRNAs, which are not translated into proteins. These ncRNAs can be categorized into short‐stranded ncRNAs and lncRNAs according to their molecular sizes, and play an important role in epigenetic regulation.^188^ They impact cellular proliferation, migration, invasion, and apoptosis through multiple pathways, significantly regulating tumor behavior and its microenvironment. Taking the Wnt/β‐catenin pathway as an example, miR‐34a serves as a key regulatory factor.^189^ It inhibits the activation of the Wnt pathway by directly targeting and degrading β‐catenin mRNA, thereby affecting the proliferation and migration of lung cancer cells. Studies have shown that miR‐34a expression is usually downregulated in lung cancer tissues, where it supports the proliferation and survival of lung cancer cells by inhibiting the Wnt pathway.^190^ Similarly, the expression of lncRNA HOTAIR is significantly increased in lung cancer, and it can promote the transcriptional activity of β‐catenin by binding to various proteins related to the Wnt signaling pathway, such as EZH2, enhancing the oncogenic effects of the Wnt pathway.^191^ In the PI3K/Akt/mTOR pathway, lncRNA GAS5 regulates apoptosis and survival of lung cancer cells by indirectly affecting the phosphorylation status of Akt through inhibiting the expression of mTOR.^192^ GAS5, acting as a tumor suppressor gene, is expressed at reduced levels in many lung cancer patients and is negatively correlated with patient prognosis.^193^

**FIGURE 4:**
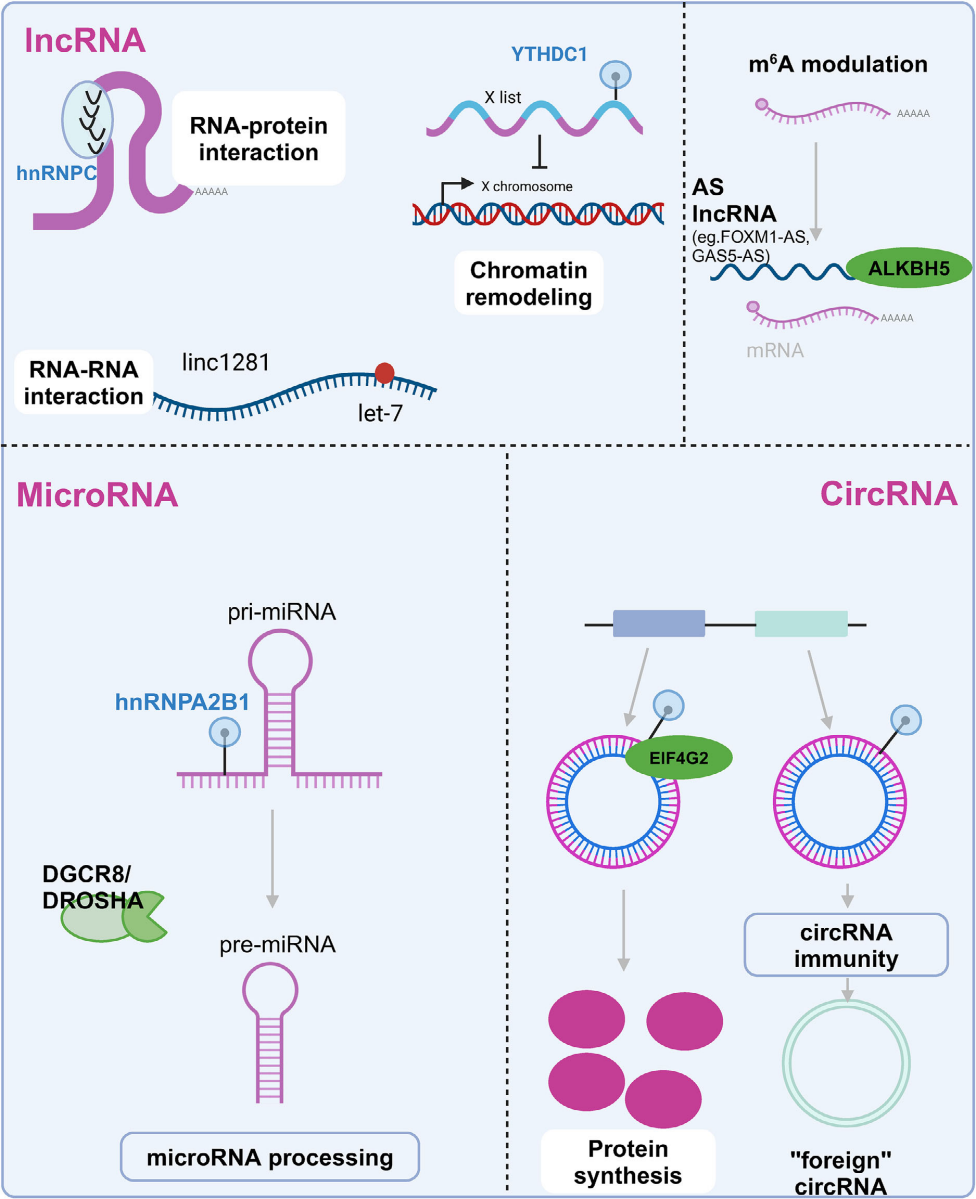
Noncoding RNA (ncRNA) regulatory mechanisms in lung cancer (including: lncRNA, miRNA, circRNA). ncRNAs are usually classified into three main categories: long noncoding RNAs (lncRNAs), microRNAs (miRNAs), and circular RNAs (circRNAs). lncRNAs interact with RNA‐binding proteins and thus have important roles in the stability and modification of mRNAs, and are also involved in chromatin remodeling, regulating the mRNA methylation status that affects translation and stability. It also participates in chromatin remodeling and regulates mRNA methylation that affects translation and stability. MicroRNAs, on the other hand, are important in posttranscriptional gene regulation from the origin of the original transcript to targeting mRNA degradation or translational repression. Cyclic RNAs are produced in reverse splicing, influence protein synthesis by sequestering RNA‐binding proteins, and regulate gene expression by acting as miRNA sponges. ncRNAs have multiple interactions in lung cancer that highlight their potential as targets for therapeutic interventions and emphasize their importance in the complex regulatory networks that influence cancer progression. Created with BioRender.com.

In terms of drug development, research into ncRNAs has shown potential therapeutic prospects. For instance, miRNA‐based therapeutic strategies, such as the development of synthetic miRNA mimetics or anti‐miRNA oligonucleotides, are currently under development to restore or inhibit the function of specific ncRNAs, regulating key pathways in lung cancer.^194^, ^195^ These therapies have demonstrated potential in preclinical studies and early clinical trials, especially for patients exhibiting specific ncRNA expression abnormalities (Figure 4).

##### Long noncoding RNAs

While lncRNAs are a type of ncRNA that do not translate into proteins, they significantly influence cellular functions. In the context of lung cancer, lncRNAs are a focal point of research, alongside miRNAs and circRNAs. lncRNAs modulate gene expression through various routes such as interacting with other RNA molecules or proteins, participating in epigenetic modifications, and affecting the structure of chromatin and gene activation.^208^ Specific lncRNAs in lung cancer act as either tumor suppressors or oncogenes. For instance, the lncRNA MALAT1 (Metastasis‐Associated Lung Adenocarcinoma Transcript 1) is often upregulated in lung cancer, where it promotes tumor growth and metastasis by influencing cell cycle genes.^209^ TUG1 (Taurine Upregulated Gene 1) expression abnormalities are associated with enhanced tumor cell proliferation, migration, and invasion in multiple cancers.^210^ Exploring lncRNA regulatory mechanisms is key to understanding lung cancer pathogenesis and progression, pointing to novel therapeutic approaches

##### Circular RNAs

circRNAs, unique in their covalently closed loop structures, do not encode proteins but are implicated in the molecular etiology of lung cancer. Their role predominantly involves acting as competitive endogenous RNAs or “miRNA sponges,” indirectly modulating gene expression and influencing oncogenic or tumor‐suppressive signaling cascades.^211^ Take ciRS‐7, a circRNA that acts as a sponge for miR‐7, altering the expression of several oncogenes and contributing to the molecular mechanisms underlying tumorigenesis. By sequestering miR‐7, ciRS‐7 upregulates target genes like EGFR, which are central to tumor development and progression.^212^ Another circRNA, CDR1as, intervenes in the regulation of lung cancer tumor suppressor factors by binding to miR‐7, thereby affecting cellular proliferation and apoptosis. The dysregulation of circRNAs, whether through aberrant expression or modification, plays a critical role in various cancer biology facets, including tumor growth, metastasis, and chemoresistance. Delving into the regulatory machinations of circRNAs unlocks potential for diagnostic and therapeutic innovations in lung cancer.^213^

##### MicroRNAs

miRNAs are small, ncRNA molecules approximately 22 nucleotides in length that play a crucial role in regulating gene expression posttranscriptionally. In lung cancer, miRNAs have emerged as significant biomarkers and therapeutic targets due to their ability to modulate key cancer‐related pathways.^214^ miRNAs regulate gene expression by binding to complementary sequences on the 3′ untranslated regions (3′ UTR) of target mRNAs, leading to mRNA degradation or translational repression. This regulation impacts various cellular processes, including cell proliferation, apoptosis, and metastasis. In lung cancer, specific miRNAs can function as oncogenes, known as “oncomiRs,” or as tumor suppressors.^215^ For example, miR‐21 is one of the most commonly upregulated miRNAs in lung cancer, acting as an oncomiR by targeting tumor suppressor genes such as PTEN and PDCD4, thereby promoting cancer progression and metastasis. Conversely, let‐7 is a well‐known tumor‐suppressive miRNA in lung cancer, which targets oncogenes like RAS and HMGA2, resulting in the inhibition of tumor growth and spread. The dysregulation of miRNA expression is directly related to the pathogenesis of lung cancer. For instance, miR‐34a, which is downregulated in various types of lung cancer, is known to affect the cell cycle and apoptosis by modulating multiple signaling pathways, such as p53.^216^ Additionally, the upregulation of miR‐155 is common in lung cancer, where it promotes tumorigenesis and progression by affecting immune responses and inflammatory processes^217^ (as summarized in Table 2).

**TABLE 2:** MicroRNAs commonly dysregulated in lung cancer.

miRNA	Expression of miRNA	Pathways/mechanisms of action	Events Influenced by its actions	References
miR‐942	Downregulation	One of the key mechanisms by which miR‐942 influences lung cancer progression is by regulating epithelial–mesenchymal transition (EMT).	Diagnostic marker for lung cancer	^196^, ^197^
miR‐601	Downregulation	Not clear	Diagnostic marker for lung cancer, associated with OS (overall survival) and RFS (relapse‐free survival)	^198^
miR‐31	Downregulation	Represses tumor suppressors LATS2 and PPP2R2A	Clonal growth of lung cancer cell sand in vivo tumorigenicity	^199^, ^200^
miR‐630	Downregulation	Inhibits p53‐regulated proapoptotic pathways	Chemotherapy resistance, specifically to cisplatin and carboplatin	^201^, ^202^
miR‐631	Downregulation	Plays crucial roles in various aspects of cancer biology, including cell proliferation, apoptosis, metastasis, and drug resistance	Diagnostic marker for early stage of lung cancer	^203^
miR‐17	Upregulation	Cooperate with cMyc to accelerate tumor development and promote tumor neovascularization	Tumor development and neovascularization	^204^
miR‐18a				
miR‐19a				
miR‐20a				
miR‐19b‐1				
miR‐92‐1				
miR‐492	Upregulation	Highly expressed in many different tumors and was associated with the process of tumorigenesis, metastasis, and recurrence	Diagnostic marker for early stage of lung cancer	^205^, ^206^
miR‐590‐3p	Upregulation	inhibited the expression of OLFM4 protein by binding to the 3′UTR site of OLFM4, which is its downstream target gene, thus promoting invasion and metastasis of lung adenocarcinoma	Diagnostic marker for early stage of lung cancer	^207^

Studying miRNAs in lung cancer helps in understanding the molecular mechanisms of lung carcinogenesis and provides a foundation for developing new diagnostic and therapeutic strategies. miRNAs play a significant role in the pathogenesis and progression of lung cancer.^218^ These small, ncRNA molecules are key regulators of gene expression and are involved in various cellular processes, including cell proliferation, differentiation, and apoptosis.^219^ In lung cancer, specific miRNAs have been found to be either upregulated or downregulated, making them potential biomarkers for early diagnosis, prognosis, and therapeutic targets.^220^

miR‐942 and miR‐601 are examples of miRNAs that are significantly increased in the serum of lung cancer patients.^221^ Their elevated levels compared with benign lung diseases and healthy individuals suggest a strong association with lung cancer. These miRNAs could serve as promising molecular signatures for the early detection and prognosis prediction of the disease.

Conversely, miR‐492 and miR‐590‐3p are also identified as differentially expressed in lung cancer.^207^ The expression patterns of these miRNAs, along with miR‐631, are distinct enough to differentiate healthy individuals from early‐stage lung cancer patients. This indicates their potential utility as serum biomarkers for the early diagnosis of lung cancer. Biomarkers for Early Diagnosis of Non‐small Cell Lung Cancer. In addition to these, miR‐31 has been studied for its broader role in various pathologies, including lung cancer. This miRNA is differentially expressed in rejecting heart allografts and in serum, and it shows a strong correlation with tissue expression. Such characteristics point to miR‐31's potential as a noninvasive biomarker. Notably, miR‐21 is often found to be overexpressed in lung cancer and is linked to the advancement of the tumor and poorer outcomes. Its engagement in a variety of molecular pathways points to its crucial importance in understanding the development of lung cancer and in crafting novel approaches for early‐stage treatment.^222^, ^223^ Conversely, miR‐34a typically exhibits reduced expression in lung cancer and acts as a tumor suppressor.^224^ Its significant influence on cell cycle control and apoptosis positions it as a promising target for therapeutic intervention.^225^

In sum, the study of miRNAs in lung cancer provides invaluable insights into the molecular dynamics of cancer. The capacity to identify and monitor these miRNAs in blood serum could pave the way for noninvasive methods for the early detection, diagnosis, and treatment of lung cancer.

## 4 EPIGENETIC REGULATION IN TME

In the lung cancer TME, epigenetic modifications play a critical regulatory role, impacting not only the characteristics of tumor cells themselves but also profoundly influencing the functionality and interactions of various cellular components within the TME,^226^ including immune cells, angiogenesis, and stromal cells. Epigenetic changes such as DNA methylation and histone modifications can regulate the expression of tumor cell surface antigens and immune regulatory molecules, significantly affecting the function of immune cells.^227^ Specifically, the upregulation of PD‐L1 (Programmed Death‐Ligand 1) enables tumor cells to inhibit T cell activity, effectively evading immune surveillance.^228^ In the TME, the methylation level of the PD‐L1 gene significantly influences its expression, directly impacting the tumor cells’ ability to escape immune detection. Low methylation in the promoter region of the PD‐L1 gene is usually associated with increased expression, enhancing the tumor cells’ ability to suppress T cell activity through binding with PD‐1 on T cells, thereby facilitating immune evasion. Furthermore, tumor cells can enhance PD‐L1 expression by activating signaling pathways such as JAK/STAT, PI3K/AKT, and MAPK in response to inflammatory factors like IFN‐γ or internal genetic alterations within the TME.^229^ Additionally, changes in the methylation of cytokine or chemotactic factor genes can affect their secretion, thereby influencing the ability of dendritic cells and macrophages to recognize and destroy tumor cells. This regulatory effect is crucial in immune checkpoint therapies, where the epigenetic status directly affects treatment outcomes (as summarized in Table 3).

**TABLE 3:** Epigenetic modification in the immune microenvironment of non‐small cell lung cancer.

TME cell type	Epigenetic modification	Mechanism	Exosome role	References
Epithelial cells	DNA methylation	Silencing of tumor suppressor genes via promoter hypermethylation, leading to dysregulated cell cycle and apoptosis pathways	Exosomes from epithelial cells carry methylated DNA that can influence neighboring cells, enhancing tumorigenic signals.	^239^
Macrophages	Histone acetylation	Acetylation of histones at cytokine gene promoters, facilitating chromatin accessibility and transcriptional activation of proinflammatory cytokines	Macrophage‐derived exosomes transfer acetylated histones to other immune cells, boosting inflammatory responses.	^240^
T cells	Histone methylation	Methylation of histone H3 lysine 4 (H3K4) at effector cytokine gene promoters, promoting transcriptional activation and effector function	T cell exosomes contain mRNA and miRNAs that regulate methylation patterns in recipient cells, affecting immune responses.	^241^
Dendritic cells	DNA hydroxymethylation	Ten‐eleven translocation (TET) enzymes catalyze hydroxymethylation of DNA, influencing antigen processing and presentation through modulation of MHC class II expression.	Exosomes from dendritic cells may carry hydroxymethylated DNA, influencing antigen presentation capabilities in other immune cells.	^242^
Fibroblasts	Histone phosphorylation	Phosphorylation of histone H2AX facilitates recruitment of DNA damage response proteins, promoting tumor microenvironment remodeling and immune evasion.	Fibroblast‐derived exosomes can modify the chromatin landscape of cancer cells, promoting DNA damage repair and tumor survival.	^233^, ^243^
NK cells	DNA demethylation	Active demethylation mediated by TET enzymes at NK cell effector gene loci, enhancing expression of cytotoxic molecules such as perforin and granzyme B	NK cell exosomes enhance cytotoxic responses in other NK cells by transferring demethylating enzymes and cytotoxic gene transcripts.	^244^, ^245^
B cells	Histone ubiquitination	Ubiquitination of histone H2A at immunoglobulin gene loci, facilitating transcriptional elongation and class‐switch recombination during antibody production	B cell exosomes carry modified histones and RNA molecules that influence antibody production in recipient B cells.	^246^
Myeloid‐derived suppressor cells	Histone deacetylation	Histone deacetylases (HDACs) repress transcription of proinflammatory genes by deacetylating histones at gene promoters, contributing to immunosuppressive phenotype.	Exosomes from these cells transfer HDACs to other immune cells, enhancing the immunosuppressive microenvironment	^247^

Regarding angiogenesis, epigenetic mechanisms regulate the expression of key factors such as vascular endothelial growth factor (VEGF), essential for tumor growth and dissemination.^230^ In the TME, the upregulation of VEGF is controlled by complex epigenetic mechanisms, including histone acetylation and DNA methylation. Specifically, histone acetylation generally enhances the transcriptional activity of certain genes by reducing the affinity of core histones for DNA, thereby making the transcriptional machinery more accessible to these genes. In the case of the VEGF gene, increased levels of histone acetylation can promote an open state in its promoter region, thereby enhancing VEGF transcriptional activity. Concurrently, DNA methylation typically acts at promoter regions to suppress gene expression. However, low methylation states in the VEGF gene promoter may facilitate its upregulation, especially under hypoxic or inflammatory conditions, which are common in tumor environments. Through these epigenetic regulations, VEGF can respond to changes in the tumor environment, promoting angiogenesis and providing the necessary nutrients and oxygen supply for tumor growth and spread.^231^ This understanding of VEGF expression regulation not only reveals how tumors adapt their microenvironment through epigenetic mechanisms but also provides potential targets for antitumor strategies aimed at the VEGF signaling pathway.^232^

In addition, epigenetic alterations can shift the phenotype of stromal cells from an antitumor to a protumor state, enhancing the tumor‐supportive behavior of the TME. In the TME of lung cancer, tumor cells are able to induce epigenetic reprogramming of surrounding stromal cells (e.g., fibroblasts) through the secretion of specific signaling molecules such as transforming growth factor‐β (TGF‐β).^233^ This reprogramming mainly involves alterations in DNA methylation and histone modifications, which affects the gene expression pattern of stromal cells. The signaling pathway activated by TGF‐β promotes the expression of MMPs genes in fibroblasts.^234^ These MMPs play an important role in the TME by not only remodeling the surrounding extracellular matrix to provide a physical pathway for tumor invasion, but also by degrading stromal components to promote tumor cell migration and spread. This epigenetic alteration and signaling pathway activation induced by tumor cells suggests that tumors are able to regulate their microenvironment to support their growth and invasion through complex intercellular communication and epigenetic mechanisms.^235^ An in‐depth understanding of this mechanism provides important information for the development of therapeutic strategies that target the TME.

In the TME, exosomes play a crucial role, especially in the communication process between tumor cells and surrounding cells. Exosomes can carry and deliver a variety of biomolecules, including miRNAs and mRNAs, which directly affect the epigenetic state and biological behavior of the recipient cells.^236^ For example, the delivery of specific miRNAs by tumor cells via exosomes can inhibit the expression of antitumor genes in the recipient cells or activate signaling pathways that promote tumor growth and invasion.^237^ This exosome‐mediated signaling not only adjusts the microenvironment around the tumor, but also creates conditions for tumor spread by altering the epigenetic landscape of distant normal or precancerous tissues. Exosomes also have a key role in immune regulation, especially in the tumor immune escape mechanism. Signaling molecules delivered by tumor cells through exosomes can directly affect the activation and function of immune cells, for example, by delivering inhibitory miRNAs or proteins to inhibit the activation and proliferation of T cells or to enhance the inhibitory function of regulatory T cells, which reduces the ability of the immune system to attack tumor cells.^238^ In addition, exosomes released by tumor cells can carry immune checkpoint proteins such as PD‐L1, which directly bind to PD‐1 on the surface of T cells and inhibit their activity, further promoting immune escape of tumor cells.

The use of epigenetic mechanisms to modulate immune cells to enhance the efficacy of immunotherapy in the lung cancer TME is a cutting‐edge strategy. By adjusting the methylation and acetylation levels of specific immune‐related genes (e.g., activation and effector molecules of T cells), it is possible to directly affect the expression of these genes, thereby altering the activity and function of immune cells. For example, the antitumor response capacity of T cells can be enhanced by decreasing the methylation level of inhibitory cytokine genes or increasing the acetylation of genes associated with promoting cell activation. In addition, epigenetic modulation targeting immune cells can not only improve their recognition and killing efficiency against tumor cells, but also reduce their depletion status, thereby improving the overall efficacy of existing immunotherapies such as immune checkpoint inhibitors. The implementation of such strategies requires comprehensive consideration of the complex interactions in the TME, as well as the need for individualized medicine to provide patients with more precise and effective treatment options.

## 5 CLINICAL APPLICATIONS AND CHALLENGES OF EPIGENETIC BIOMARKERS

In the realm of oncology, epigenetic biomarkers, encompassing DNA methylation, histone modifications, mRNA expression, and exosomal contents, have shown significant promise for enhancing cancer diagnosis, prognosis, and therapeutic strategies. Currently, numerous drugs targeting epigenetic modification markers have been developed for clinical use (as summarized in Table 4). However, DNA methylation remains most closely associated with liquid biopsy.^248^ DNA methylation patterns serve as early detection markers in liquid biopsies, allowing for the identification of cancer through noninvasive means. Furthermore, the methylation status of tumor suppressor genes offers prognostic value, correlating with disease severity and patient survival outcomes.^249^ Histone modifications provide insights into cancer subtypes and progression, facilitating personalized treatment approaches, while specific enzymes responsible for these modifications, such as HDACs and HATs, have emerged as potential therapeutic targets.^250^ mRNA levels reflect tumor cell activity and can be used to monitor disease progression and response to treatment. Exosomes play a crucial role as carriers of a diverse array of biomolecules, including DNA, RNA, and proteins, offering a window into the tumor's molecular state through easily accessible body fluids, thus aiding in the tracking of cancer progression or therapeutic response.

**TABLE 4:** Lung cancer epigenetic drugs approved or under clinical trials.

Epigenetic target	Drug type	Approved drugs and conditions	Drugs in clinical trials	References
DNA methylation	DNMT inhibitors	Azacitidine (for myelodysplastic syndrome, off‐label in lung cancer)	Guadecitabine (in Phase II for NSCLC)	^256^
Histone acetylation	HDAC inhibitors	Vorinostat (for cutaneous T cell lymphoma, off‐label in lung cancer)	Romidepsin (in Phase II for NSCLC)	^159^
Histone methylation	EZH2 inhibitors	Tazemetostat (for epithelioid sarcoma, in trials for NSCLC)	Tazemetostat (in Phase II for NSCLC)	^257^
BET proteins	BET inhibitors	Not clear	CPI‐0610, OTX015 (in early phase trials for NSCLC)	^258^
Multiple epigenetic marks	Multiacting agents	Panobinostat (for multiple myeloma, in trials for NSCLC)	Mocetinostat (in Phase II for NSCLC)	^259^

The integration of DNA methylation analysis with liquid biopsy technology marks a transformative approach in the realm of oncology, providing a robust method for early cancer detection and continuous monitoring. DNA methylation, which entails the addition of a methyl group primarily to CpG dinucleotides in DNA, alters gene expression without changing the DNA sequence itself. This epigenetic modification, especially the hypermethylation of tumor suppressor genes, can contribute to oncogenesis by silencing critical genes. Liquid biopsy capitalizes on this by detecting ctDNA in body fluids like blood, which retains the genetic and epigenetic characteristics of the original tumor, including methylation patterns.^251^ This noninvasive technique allows for the early detection of cancer through simple blood samples, potentially before the tumor is clinically evident or detectable through conventional imaging.

Furthermore, DNA methylation markers in ctDNA offer enhanced specificity and sensitivity in cancer screening, significantly improving early detection and the ability to monitor disease progression or response to treatment.^252^ For instance, the methylation status of genes such as SEPT9 in colorectal cancer screening has demonstrated higher sensitivity than traditional fecal occult blood tests.^253^ Early detection through these markers has been shown to improve survival rates, as evidenced by studies in cancers such as pancreatic, ovarian, and lung cancer. Additionally, changes in methylation patterns during therapy can provide real‐time insights into treatment efficacy and disease status, aiding in personalized treatment adjustments and improving overall management of the disease. These advances highlight the critical role of epigenetic biomarkers in enhancing diagnostic accuracy, monitoring treatment response, and ultimately improving outcomes for cancer patients.

Despite the significant potential of epigenetic biomarkers, their clinical application faces several challenges. Currently, there is a lack of standardized methods for the extraction and analysis of these biomarkers, particularly exosomes and histone modifications, which impacts the consistency and reproducibility of the data. The sensitivity and specificity of biomarkers can vary greatly among different cancers and their subtypes, making it challenging to identify biomarkers that are both sensitive and specific.^254^ Interpreting epigenetic data require specialized knowledge, and translating these findings into clinical practice necessitates further validation and clinical trials. Additionally, ethical and privacy concerns related to the collection and use of patients' genetic and epigenetic information require stringent regulations and guidelines to ensure data protection and ethical compliance.^255^ Moreover, epigenetic inhibitors may have adverse effects on the TME. The TME is not uniformly affected by single epigenetic inhibitors. For instance, inhibitors like HDAC inhibitors may have varying effects on different immune cells. ACY‐1215 targets HDAC6 (a class IIb HDAC) while preserving HDAC9 activity, which promotes positive regulation of antitumor immunity. Thus, more in‐depth epigenetic research is needed to clarify the regulatory mechanisms induced by various epigenetic modifications, thereby developing highly selective epigenetic inhibitors for more effective targeting.

## 6 CONCLUSIONS AND OUTLOOK

In the context of early‐stage lung cancer, a variety of epigenetic mechanisms play crucial roles in tumor progression. These mechanisms include DNA methylation, histone modifications, the TME, mRNA dynamics, and ncRNAs such as miRNAs, circRNAs, and lncRNAs.

DNA methylation plays a crucial role in suppressing gene activity and maintaining genomic stability by inhibiting recombination events between repetitive sequences. This epigenetic modification often results in the silencing of inhibitory factors or the increased expression of activating factors, which is a common mechanism for the abnormal activation of the Wnt pathway. For example, methylation of the promoter regions of genes such as those in the DKK family (e.g., DKK1, DKK3), the SFRP family (e.g., SFRP1, SFRP2), and WIF1 (Wnt Inhibitory Factor 1) leads to their downregulation or silencing, thereby lifting the inhibition on the Wnt pathway and promoting tumor cell proliferation and survival.^260^ In some cases, the positive regulators in the Wnt signaling pathway may be activated due to hypomethylation or other mechanisms, increasing the expression and activity of β‐catenin, which sustains the activation of the Wnt signaling pathway, further promoting the proliferation, invasion, and metastasis of tumor cells. Given the regulatory impact of DNA methylation on the Wnt signaling pathway, drugs targeting DNA methylation, such as DNMT inhibitors (e.g., Azacitidine and Decitabine),^261^ may be effective in inhibiting tumor progression. Research is also exploring how to reverse these methylation changes to restore normal regulation of the Wnt signaling pathway.

Histone modifications represent another significant aspect of epigenetic regulation, affecting cancer gene expression through mechanisms such as acetylation and deacetylation, which alter chromatin structure and thereby influence DNA accessibility and transcriptional activity. Acetylation modifications, by opening up chromatin structure, facilitate the binding of transcription factors to promoter regions, thus promoting gene expression. This mechanism leads to the sustained activation of the Wnt pathway in lung cancer cells, thereby promoting tumor proliferation and aggressiveness. Histone acetylation is typically associated with the activation of gene expression, such as the transcriptional activity of β‐catenin encoded by CTNNB1, a key component of the Wnt pathway.^262^ Conversely, histone deacetylation usually results in gene silencing, potentially lowering the transcriptional activity of Wnt pathway inhibitory genes, such as those in the DKK and SFRP families, thus lifting the inhibition on the Wnt pathway. At the same time, HDACs play a crucial role in this process; HDAC inhibitors like Vorinostat, by blocking deacetylation, may reactivate these inhibitory genes, thereby countering the abnormal activation of the Wnt signal.^263^ Methylation also plays a role in histone modifications; for example, if H3K9 methylation occurs in the promoter regions of Wnt pathway inhibitory genes, it may lead to gene silencing, resulting in the overactivation of the Wnt signaling pathway. Conversely, an increase in H3K4 methylation may promote the expression of genes that activate the Wnt pathway, further driving the progression of lung cancer.^264^ Due to the significance of histone modifications in regulating the Wnt signaling pathway, drugs targeting histone‐modifying enzymes, including HDACs, HATs, and HMTs, are considered to have potential therapeutic value. For example, HDAC inhibitors can partially reverse the abnormal activation of the Wnt signaling pathway by restoring the expression of Wnt inhibitory factors, thereby inhibiting the progression of lung cancer.

ncRNAs play a critical regulatory role by finely tuning the expression and activity of specific factors within key signaling pathways. These ncRNAs, including miRNAs, lncRNAs, and circRNAs, significantly regulate tumor behavior and its microenvironment by simultaneously affecting cellular processes such as proliferation, migration, invasion, and apoptosis. Taking the Wnt/β‐catenin pathway as an example, miR‐34a is a key regulator that inhibits the activation of the Wnt pathway by directly targeting and degrading β‐catenin mRNA, thereby affecting the proliferation and migration of lung cancer cells.^265^ Research shows that miR‐34a is typically downregulated in lung cancer tissues, and by inhibiting the Wnt pathway, it supports the proliferation and survival of lung cancer cells. Similarly, the expression of lncRNA HOTAIR is significantly increased in lung cancer, and it can promote the transcriptional activity of β‐catenin by binding to various proteins associated with the Wnt signaling pathway, such as EZH2, thereby enhancing the oncogenic effects of the Wnt pathway.^266^ In the PI3K/Akt/mTOR pathway, lncRNA GAS5 regulates the apoptosis and survival of lung cancer cells by indirectly affecting the phosphorylation state of Akt through inhibiting the expression of mTOR.^267^ GAS5 acts as a tumor suppressor gene, and its expression is reduced in many lung cancer patients, negatively correlating with patient prognosis. In terms of drug development, research into ncRNAs has shown potential therapeutic prospects.^268^ For example, miRNA‐based therapeutic strategies, such as the development of synthetic miRNA mimetics or anti‐miRNA oligonucleotides, are currently being developed to restore or inhibit the function of specific ncRNAs, regulating key pathways in lung cancer. These therapies have shown potential in preclinical studies and early clinical trials, especially for patients with specific ncRNA expression abnormalities.

In the field of early‐stage lung cancer diagnostics, the combination of liquid biopsy techniques and the detection of epigenetic modification markers has significantly enhanced the accuracy and feasibility of early detection. Liquid biopsy is a noninvasive approach that analyzes ctDNA, cell‐free DNA, and other biomarkers in blood to detect cancer potentially up to 4 years before abnormalities are visible through conventional imaging studies. Specifically, epigenetic alterations, such as DNA methylation, precede common genetic mutations in the development of lung cancer. Research on the methylation of the SHOX2 gene indicates a significant elevation in the methylation levels among lung cancer patients, which can be detected through blood samples and thus serve as an early diagnostic marker for asymptomatic high‐risk populations. Moreover, the analysis of methylation profiles in patient blood has successfully identified lung cancer in advance, demonstrating the potential of liquid biopsy techniques in preclinical studies. The use of composite epigenetic biomarkers, including specific patterns of DNA methylation and histone modifications, effectively distinguishes between healthy individuals and lung cancer patients. These instances illustrate that integrating liquid biopsy with the detection of epigenetic modifications not only offers an efficient strategy for the early diagnosis of lung cancer but also aids in understanding the pathogenesis of the disease and in developing new treatment approaches. However, epigenetic modulators may adversely affect the TME. Despite the development of numerous drugs targeting epigenetic factors, epigenetic inhibitors could potentially have detrimental impacts on the TME, as TME is not uniformly affected by a single epigenetic inhibitor. For instance, HDAC inhibitors might differentially impact various immune cells. ACY‐1215 targets HDAC6 (Class IIb HDAC) while preserving HDAC9 activity, promoting positive regulation of antitumor immunity. Importantly, the mechanisms of action of epigenetic regulatory factors are complex; in cancer, they may need to maintain the expression of some key target genes. If these regulators are broadly targeted, the balance could be disrupted enough to lead to cellular catastrophe. Therefore, further in‐depth epigenetic research is necessary to delineate the characteristics of various epigenetic modifications in tumor regulation, thereby developing highly selective epigenetic inhibitors for more effective targeting efficiency.

## AUTHOR CONTRIBUTIONS

*Writing—original draft; conceptualization; visualization*: Lingfeng Bi. *Supervision*: Xin Wang and Jiayi Li. *Writing—review and editing; project administration; supervision; funding acquisition*: Zhoufeng Wang. *Writing—review and editing; project administration; supervision; funding acquisition*: Weimin Li. All authors have read and approved the article.

## CONFLICT OF INTEREST STATMENT

The authors declare no conflicts of interest.

## ETHICS STATEMENT

Not applicable.

## Data Availability

Not applicable.
